# Prioritizing syphilis control: Now is the time for action

**DOI:** 10.3389/fmed.2022.899200

**Published:** 2022-08-22

**Authors:** Joseph D. Tucker, Gifty Marley, Michael Marks, David Mabey

**Affiliations:** ^1^Institute for Global Health and Infectious Diseases, University of North Carolina at Chapel Hill, Chapel Hill, NC, United States; ^2^Clinical Research Department, Faculty of Infectious and Tropical Diseases, London School of Hygiene and Tropical Medicine, London, United Kingdom; ^3^SESH Global, Guangzhou, China; ^4^Hospital for Tropical Diseases, University College London, London, United Kingdom

**Keywords:** syphilis, advocacy, COVID-19, partner services, crowdsourcing

## Abstract

Syphilis control programs and research received fewer resources and attention compared to HIV and other sexually transmitted infections (STIs) in the pre-pandemic era. The neglect of syphilis within comprehensive STI control efforts may be related to diagnostic (poor diagnostics), historical (legacies of racism in research), public health (limited partner services), and social problems (limited public engagement). At the same time, there are increasingly compelling reasons to prioritize syphilis control programs and research by harnessing lessons learned and advances during COVID-19. The closure of many STI facilities has accelerated new syphilis diagnostic pathways (e.g., syphilis self-testing), providing new ways for people to be screened outside of clinics. COVID-19 has underlined health inequities that fuel syphilis transmission, providing an opportunity to reckon with the historical legacy of racism that is linked to syphilis research. COVID-19 partner tracing efforts have also contributed to additional resources for partner services which may enhance syphilis control efforts. Finally, COVID-19 has demonstrated the importance of public engagement, making the case for greater public involvement in syphilis control and prevention programs. Urgent action is needed to prioritize syphilis control in a wide range of settings.

## Introduction

Prior to COVID-19, syphilis was often neglected in global health research and programs. According to an analysis of data on infectious diseases research supported by G20 (a group of twenty countries) countries across 18-years, syphilis received the least amount of research grants per disability-adjusted life year (1). However, syphilis increases the risk of acquiring and transmitting HIV infection (2). Syphilis infection among pregnant women increases the risk of neonatal death, preterm labor, and other adverse birth outcomes (3). In addition, there is substantial stigma associated with syphilis (4). Improving syphilis services could decrease stillbirths, decrease syphilis-related stigma, decrease persistent health disparities related to at-risk groups, and improve the lives of many vulnerable individuals.

Ultimately, repeated calls to action from academic researchers and policy-makers (5) have not resulted in meaningful policy change. Yet the COVID-19 provides a new opportunity to re-think syphilis control because both are infectious diseases that require partner services, have self-testing options, and exacerbate health inequalities. This policy perspective examines the relative neglect of syphilis within comprehensive STI control systems, assessing critical historical, diagnostic, public health, and social issues before COVID-19. We also map out concrete ways that COVID-19 interventions could be used to prioritize syphilis control within health systems.

## Malign neglect

**Figure 1:**
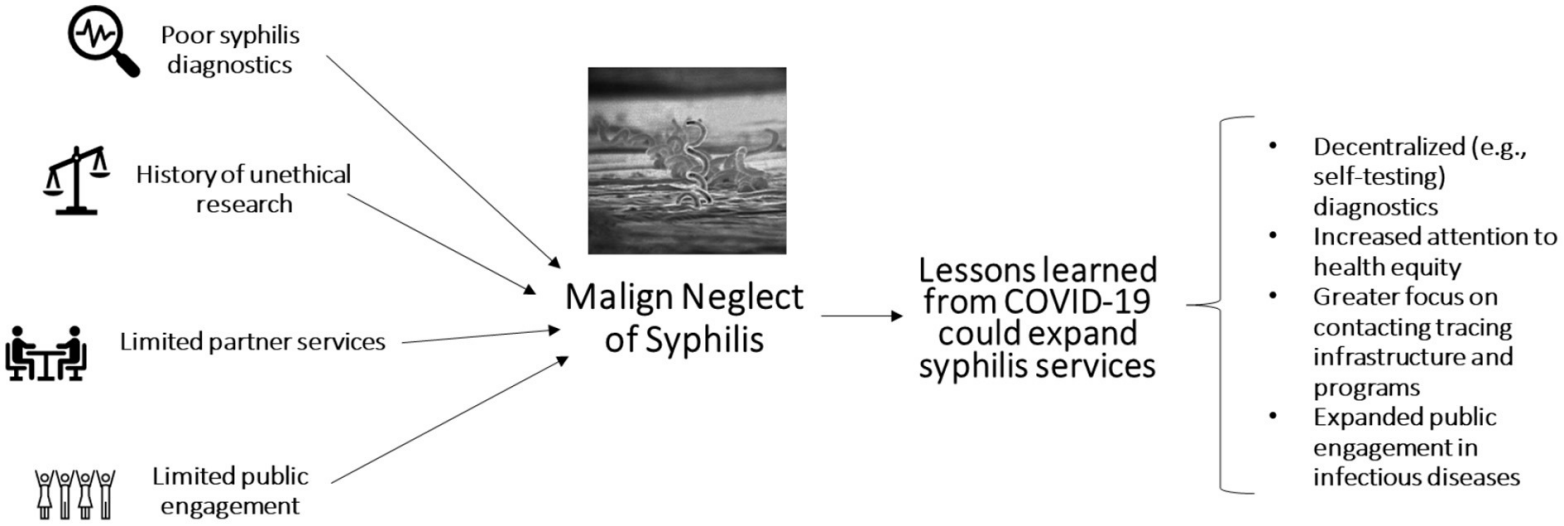
Overview of the malign neglect of syphilis within comprehensive STI control programs.

The prevailing approach to syphilis control efforts within public health systems may be characterized as one of malign neglect—causing harm by doing nothing. Routine syphilis screening rates are low in many countries (6). The malign neglect of syphilis within comprehensive STI control efforts may be related to historical issues, diagnostics, partner services, and social issues (Figure 1).

The unique history of syphilis research has cast a long shadow on subsequent syphilis research. For example, in the United States, the Tuskegee trial enrolled poor, black men who were intentionally misled about the research study and denied treatment (7). An analysis of US Centers for Disease Control mortality data found that disclosure of the Tuskegee mistreatment in 1972 was correlated with greater mortality, medical mistrust, and delayed care health care seeking among black men in the United States (8). The Tuskegee mistreatment of black men and related inequalities may partly explain the higher burden of syphilis among black men at the time (9). Similar unethical syphilis research was organized by US government scientists in Guatemala (10) and these unethical trials have helped to inform human subjects training. The history of unethical syphilis research may discourage investigators from focusing on syphilis and research participants from joining syphilis studies.

Until recently, poor syphilis diagnostics have been another major barrier to expanded syphilis research in many settings. Syphilis diagnostics remained largely unchanged over the course of the 20th century (11). The most commonly used non-treponemal test (rapid plasma regain, RPR) required equipment, reagents, and training that many resource-constrained clinics lacked (12). Centralized syphilis testing at clinics made it difficult for many key populations to receive regular syphilis testing. In recent years, affordable, sensitive and specific point of care serological tests (13) have become available using the lateral flow format. This requires no equipment and has been widely used by the general public for SARS-CoV2 self-testing, opening new opportunities for syphilis screening in resource-limited settings (14).

Partner notification and testing services are essential components of a comprehensive syphilis response, but have historically been constrained by limited financial resources. Partner services include identification, testing, and treatment of sexual partners of confirmed syphilis cases. Syphilis transmission rates are extremely high (51–64% per sexual partnership) (15), underlining the importance of timely partner services. Incomplete partner services before COVID-19 have thwarted syphilis control programs. Syphilis partner services are chronically under-funded within many public health departments (16), contributing to incomplete partner service programs for early syphilis cases (17).

Finally, public engagement in syphilis research has been under-developed. Public engagement refers to a mutually beneficial interaction between specialists and non-specialists to develop solutions. Whereas, HIV control benefitted from strong public engagement in research dating back to the 1980s (18, 19), syphilis has not inspired widespread public engagement programs. There have been fewer public engagement programs to engage local communities about syphilis infection compared to HIV. Public health HIV screening programs have generally not been integrated with syphilis screening programs in many health systems (5), despite strong public engagement focused on other STIs.

## COVID-19 and syphilis

Lessons learned from COVID-19 have the potential to transform sexual health service delivery systems, including syphilis-specific strategies globally. Despite the lack of direct links between COVID-19 interventions and syphilis, there are several COVID-19 developments that could indirectly help to prioritize syphilis in broader STI control research and programs. Programs and research focused on COVID-19 inequities, diagnostics, partner services, and public engagement may enhance syphilis projects during and beyond the COVID-19 pandemic.

First, COVID-19 has accelerated public health systems for decentralized diagnostic testing, including self-testing, self-sampling, and community-based testing. Self-testing involves a person conducting and interpreting their own test result. Clinic closures and travel restrictions during COVID-19 have accelerated syphilis self-testing uptake (20, 21) and expanded self-sampling pilots (22). Expanding these opportunities for syphilis testing in diverse settings could help to catalyze more syphilis control programs, policies, and research.

Second, COVID-19 has highlighted the impact of inequities in delivering health services. Many COVID-19 programs have explicitly focused on better serving the needs of ethnic and racial minorities. Racial disparities in COVID-19 vaccine uptake have increased urgency to rebuild trust in care providers (23). While these are necessarily long-term efforts, the renewed attention on health equity could help galvanize trust in medicine among people at greater risk for syphilis.

Third, COVID-19 pandemic has increased attention to the science and logistics of contact tracing which could help syphilis contact tracing. Responses to the COVID-19 pandemic have bolstered local public health infrastructure, especially providing resources for contact tracing, rapid testing, and related components of partner services. In some settings, the pandemic response has also altered in-person partner services toward digital adaptations. For example, a syphilis outbreak investigation during COVID-19 organized by a local health department was conducted entirely online (24).

Finally, COVID-19 provides an opportunity to strengthen public engagement in infectious diseases service delivery. Community-based coalitions that include diverse groups have formed in response to COVID-19 and could be adapted for syphilis responses (25, 26). In addition, innovative methods for public engagement such as crowdsourcing have helped to inform COVID-19 programs (27) and demonstrated to be effective in randomized controlled trials (28). Crowdsourcing has a group of people solve all or part of a problem and then implement selected solutions (29). Crowdsourcing methods have been used to increase syphilis test uptake (30, 31).

## Discussion

As COVID-19 restrictions are lifted and there is additional scope for sexual health programs, syphilis deserves greater attention. This greater focus has implications for research, implementation, and policy. From a research perspective, increased public health research on decentralized testing pathways that could be implemented in diverse settings is needed. Many people at greatest risk of syphilis do not attend centralized clinics where serological syphilis testing is available. From an implementation perspective, more intensive programs to support the dual elimination of HIV and syphilis among pregnant women through service integration is warranted. This aligns with the WHO call for the elimination of mother-to-child transmission of HIV and syphilis. From a policy perspective, partner services need to be more completely transitioned into the digital age. While COVID-19 has supported pilots, research and policies are needed.

This perspective also underlines the need for *action* at several levels of the public health system. Within local public health programs, syphilis self-testing could provide a new service delivery mechanism that does not require laboratory equipment or trained staff. Syphilis self-testing could be used in many remote settings and spur follow-up testing. At national ministries of health, more financial resources for syphilis control programs will be essential for strengthening control responses. Finally, at the global level, innovative programs to encourage syphilis testing and linkage to clinical care are necessary. Now is the time for action on syphilis.

## Ethics statement

Written informed consent was obtained from the individual(s), and minor(s)' legal guardian/next of kin, for the publication of any potentially identifiable images or data included in this article.

## Author contributions

JT wrote the first draft. All authors contributed to the manuscript and read and agreed with the final manuscript.

## Funding

This research was supported by the NIH (NICHD UH3HD096929, NIAID K24AI143471, and NIAID R01AI158826).

## Conflict of interest

The authors declare that the research was conducted in the absence of any commercial or financial relationships that could be construed as a potential conflict of interest.

## Publisher's note

All claims expressed in this article are solely those of the authors and do not necessarily represent those of their affiliated organizations, or those of the publisher, the editors and the reviewers. Any product that may be evaluated in this article, or claim that may be made by its manufacturer, is not guaranteed or endorsed by the publisher.
